# The Influence of Curator Goals on Collections of Lived Experience Narratives: A Qualitative Study

**Published:** 2021

**Authors:** Caroline Yeo, Stefan Rennick-Egglestone, Victoria Armstrong, Marit Borg, Ashleigh Charles, Laurie Hare Duke, Joy Llewellyn-Beardsley, Fiona Ng, Kristian Pollock, Scott Pomberth, Rianna Walcott, Mike Slade

**Affiliations:** 1 University of Nottingham, UK; 2 Disability North, Newcastle, UK; 3 University of South-Eastern Norway; 4 NEON Lived Experience Advisory Panel, UK

**Keywords:** Lived Experience, Curation, Narratives, Stories, Mental Health Recovery

## Abstract

**Objective:**

The aim of this study was to investigate how curator goals influence the design of curation processes for collections of mental health lived experience narratives. The objectives were (1) to characterize the goals of a range of curators of existing collections, and (2) to identify specific working practices impacted by these goals.

**Research Design and Methods:**

Thirty semi-structured interviews were conducted with a purposive sample of curators of collections of lived experience narratives. Thematic analysis was conducted. Goals and impacts on working practice were tabulated, and narrative summaries were constructed to describe the relation between the two.

**Results:**

Curators interviewed were from seven countries (Brazil, Canada, Hong Kong, India, Italy, UK, USA), and 60% had lived experience of mental health service usage. Participants discussed eight goals that inspired their work: fighting stigma, campaigning for change in service provision, educating about mental health and recovery, supporting others in their recovery journey, critiquing psychiatry, influencing policy, marketing health services, and reframing mental illness. These goals influenced how decisions were made about inclusion of narratives, editing of narrative content, withdrawal rights, and anonymization.

**Conclusions:**

Our work will support the development of curatorship as a professional practice by shaping training for curators, helping curators reflect on the outcomes they would like to achieve, and helping individuals planning a collection to reflect on their motivations. We argue that transparency is an essential orientation for curators. Transparency allows narrators to make an informed choice about donating a narrative. It allows policy makers to understand the influences on a collection and hence treat it as a source of collective evidence.

## Introduction

Lived experience narratives have been central to the recovery movement since its inception. Lived experience narratives are the first-person accounts of people experiencing mental health problems. They have served many purposes, such as inspiring others in their recovery journey,^1^ promoting collective action,^2^ or promoting stigma reduction through providing for intergroup contact.^3^ A recent systematic review on the impact on others of lived experience narratives describing recovery from mental health problems (where the term “recovery” was broadly defined) has found positive impacts, such as improving understanding of recovery and reducing of stigma.^4^ A second systematic review of work looking at the characteristics of recovery narratives has found that many reference wider social and political elements.^5^

Woods and colleagues posed the question in relation to the recovery narrative: “What might be opened up, revealed, or foreclosed in telling a recovery narrative in the first-person plural?”^6^ Their research furthers the debate around how to think about recovery at both an individual and a collective level. Narratives as they have been used in the mental health system are often individual, i.e., first-person singular. This may reflect assumptions within the system. However, collective narratives might be better placed to both capture commonalities of experience while ensuring primacy is given to lived experience.^7^ Individual stories can join together to form collective stories, which can effect actual change in the world.^8^ One mechanism for creating collective narratives might be to group related narratives into a coherent collection.

A wide range of collections of lived experience narratives have already been created, sometimes presented in books, online, or in physical exhibitions.^4,9^ In previous works, we have called the people in charge of this process ”curators,” in keeping with the common usage of this term within cultural heritage and museology.^10,11^ The curator is in charge of a number of processes such as the collection, selection, and organization of narratives.^11^ Curators of collections of lived experience narratives are an important population to explore as curators can exercise decision-making power that shapes the content of collections, and hence the collective knowledge about mental health and recovery that they present. As such, the decisions made by curators need to be understood by people working with narrative collections.^11^

To understand the decisions made by curators, we first conducted a systematic review, which developed a preliminary framework of decisions made by curators around narrative collections. The review included only publicly available documents.^9^ It was extended by an interview study, which developed a typology of decisions curators make in the process of curating a collection, identified as Values and motivation; Organization; Inclusion and exclusion; Control and collaboration; Ethics and legal; and Safety and well-being (VOICES).^11^

Both of these works identified the curation of collections as a purposeful act, and they identified that curators can be motivated by broad goals, such as influencing society or providing benefits to recipients.

If curation is a purposeful activity, then we might seek to understand the goals of curators as a broader framework within which the work of curation is negotiated and made meaningful. The aim of the current study was therefore to extend our prior work by investigating how the goals of curators influenced the curation of collections of lived experience narratives. The objectives of the current study were (1) to characterize the goals of a range of curators of existing collections, and (2) to understand specific working practices impacted by curator goals.

## Research Design and Methods

This research was undertaken as part of the Narrative Experiences Online (NEON) study (information at http://www.researchintorecovery.com/neon) between December 2018 and March 2019. NEON is a Programme Grant for Applied Research funded by the National Institute for Health Research in the UK. Ethical approval was obtained in advance from the Nottingham 2 Research Ethics Committee (reference 17/EM/0401). All participants provided written informed consent. A secondary analysis was conducted through thematic analysis to complement a previously published primary analysis.^11^ Work presented in this paper has informed three clinical trials (ISRCTN11152837, ISRCTN76355273, ISRCTN63197153).^12^

### Participants

The inclusion criterion for participants was experience of curating one or more collections of narratives, including lived experiences of either mental health service use, recovery, or mental distress. The collection could be online (e.g., a collection of videos), in print (e.g., books), or art exhibitions, with included narratives containing written, spoken, film- or image-based content. A maximum variation sample^13^ of curator lived experience of using mental health services was constructed to capture service-user curator perspectives. Curators were recruited from multiple countries and continents so as to capture country-specific social, cultural, and political contexts. Over half of the participants came from the UK, where snowball sampling was used to support recruitment to interview. Participants in the first round of interviews introduced others from their network to the study.

### Procedures

Semi-structured interviews were conducted with 30 curators, either in person (N=4), by phone (N=17), video call (N=8), or, if no other option was available, by email (N=1). A demographics form was used to capture curator background. Interviews were audiotaped and transcribed with pseudonymization, and the email interview was also pseudonymized. Interviews lasted 60–90 minutes. The cumulative length of written responses in the single email interview was five A4 pages.

### Analysis

All transcripts were coded thematically^14^ in QSR International NVivo 12 by CY. One transcript was second coded early in the process by an experienced analyst (KP) to aid in the establishment of an initial coding framework.

Coding began by identifying transcript fragments describing goals (reasons curators have for putting together a narrative collection) and their impact on working practices (how the collection is curated). Analysis of this material proceeded through constant comparison.^15^ Codes developed through this process were a mixture of a priori codes corresponding to categories previously identified in the VOICES framework,^11^ codes derived from terminology used by participants, and categories identified inductively by analysts. This was a hybrid inductive-deductive approach designed to extract as much richness as possible from the available data.

To succinctly capture relationships between goals and working practices and hence to enable contributions by analysts not directly involved in the coding process, a narrative summary of all relevant transcript content was written after each transcript was coded. Narrative summaries were discussed with other analysts as they were produced, and these discussions frequently led to refinements being made to the coding process, for example, by updating names of code. Narrative summaries were iteratively refined as analysis progressed.

A final conceptual framework of goals and impacts on working practice was produced by iteratively refining the names of nodes to most effectively represent coded content. For each goal, a narrative summary was produced to illustrate what had been learned about its impact on working practices.

## Results

Thirty interviews were conducted with curators of art exhibitions (N=8) and book/online narrative collections (N=22). Table 1 presents the sociodemographic characteristics collected.

### Goals of curators

Some participants described more than one goal that influenced their curatorial decision-making. The mean number of goals identified per participant was 1.5. Table 2 shows the number of participants identified with each goal.

### Impact on curatorial practice

Table 3 summarizes all curatorial practices that were influenced by curator goals.

### Goals of curators and impact on curatorial practice

#### Fighting stigma

Fighting stigma around mental health experiences emerged as the most important goal for participants. Two curators were interested in stigma around schizophrenia, and nine were interested in stigma about general mental health issues.

“I think we really also produced it as part of health literacy to reduce stigma, to help people see themselves and realize that there is hope and there are multiple goals now, but I think one of the early goals was definitely to change some of the landscape of how decisions are made around mental health (Participant #17).”

A goal of fighting stigma typically led to the design of curation processes, which ensured that the content of included narratives fit the anti-stigma message. For some, narratives were edited to promote fighting stigma, such as avoiding content that showed an illness narrative, focusing on a life history narrative, or focusing not on diagnosis but on the person as a human being. In some instances, narrators were not anonymized because participants felt that using a person’s name reduced stigma and hence reinforced an antistigma message. They thought that not using their name was saying that the person had something to be ashamed of.

#### Campaigning for change in service provision

The goal of some participants was to campaign for change in how mental health services were provided:

“So, I think for me, you know, the project is also an alternative way of campaigning and trying to fight all of the time, actually kind of just show, kind of lead by example, and show that there's another way and that it can work and inspire those within the system who are trying to change it and give them solidarity as well (Participant #26).”

Curatorial practices were influenced by this goal as only narratives which fit a specific political standpoint were included in the collection and other narratives were excluded. These narratives had to fit with the political message of the campaign. One participant felt that their collection was part of a campaigning movement, and she did not generally allow the withdrawal of narratives as she felt this would damage the campaign. The withdrawal of narratives would mean less content on their website and less voices supporting their campaign. In addition, this participant believed that not anonymizing the narratives gave them more power and added credibility for the overall promotion of the campaigning message of the collection; therefore, narratives were not anonymized. This was a discrepant viewpoint as all other participants allowed for possible withdrawal and anonymization.

#### Educating about mental health and recovery

Education was another goal for participants as they wanted to increase understanding and knowledge of mental health and recovery in the general public and mental health workers. Some participants wanted to educate the public to better understand what it was like to live with a mental health issue. Some participants wanted to educate the public about what it is like to go through the experience of recovery. They also wanted to educate mental health workers so that they would know how to support people on their recovery journey better and improve their practice.

“The objectives were to educate others about the experience and distress and what it feels like from inside, to pass on any insight to what was helpful or unhelpful in their experience, which could be used by others to make their experience less painful or by staff to change their practice (Participant #05).”

#### Supporting others in their recovery

Another goal was to support others in their recovery journey and offer them hope. The aim for the collection was to help other people, and this had an impact on the type of stories that were chosen to go into the collection. Curators had an aim of selecting inspirational stories for publication. These were operationalized as those that ended hopefully and positively.

“I think that the intention was it’s a lot of positive stories that are going to help people (Participant #01).”

#### Critiquing psychiatry

Another goal of participants was to critique psychiatry. One participant described how they felt part of a broader movement of critical psychiatry and that psychiatry needed reform.

“I would say the broader movement, we consider ourselves to be a critical psychiatry website, and so that's the movement that we are trying to be part of, but a lot of the people that write for us, and especially a lot of the commenters, are anti-psychiatry; they are just straight out entirely that their profession cannot be reformed or redeemed; it needs to be abolished, and so that's part of the broader movement; but what we are really trying to do is rethink psychiatry, and rethinking involves reform … The idea that psychiatry just needs to change (Participant #30).”

For all participants, only narratives that were critical of psychiatry were included in the collection.

#### Influencing policy

Two participants wanted to influence mental health policy with the work that they do, and narratives that the participant thought could create mental health policy change were chosen in the collection.

“I would say it came out of a little bit of that movement of trying to nurture first-person voice to, you know, probably to influence policy you have to say; probably it came out of a policy department (Participant #17).”

#### Marketing health services

One collection was created with the aim to promote a mental health service. The curator made a marketing booklet for a mental health team with a selection of narratives that promoted their mental health service. Narratives chosen for the booklet all showed the service in a positive light with positive stories about how the service had helped them in their recovery journey.

#### Reframing mental illness

One participant wanted to reframe mental illness experiences as transformational crises and decided to build a collection of stories that fitted this alternative paradigm.

“So, the aims of the campaign are to relieve people of the distress associated with transformational crises by offering authentic examples of personal stories and resources to engender hope and initiate recovery … and to decrease stigma, improve well-being, and influence saving of lives through providing a more compassionate and positive conceptual framework for emotional distress. So, it’s about normalizing, it’s about empowering, it’s about reducing stigma, providing hope, giving people permission to seek alternative support to the dominant mental health paradigm, but whatever works for them really (Participant #19).”

Stories were included and edited to fit this alternate view on mental health crises.

## Conclusions

### Relationship to prior work

The VOICES typology^11^ has identified the decisions that curators make during their curatorial practice. The current work has revealed the underlying goals that influenced their decision-making and identified the impact of these goals on some aspects of decision-making. Understanding the goals of curators is an established component of museology research, where curator goals are already recognized as having an important influence on the decision-making process,^16^ and our work confirms the importance of understanding curators’ goals of collections of lived experience narratives. While our focus has been on collections incorporating lived experiences of mental health problems, our work might be relevant to collections including other lived experiences, such as those of cancer survivorship^17^ and chronic pain.^18^

Fighting stigma was a central goal of curators, and the lived experience narrative has placed a central role in large scale anti-stigma programs such as by Time to Change.^19^ According to the disability activist, scholar, and author Eli Clare, “Psychiatric survivors can use our stories to change the world.”^2^ This ambition was shared by participants in our study, who had goals to campaign for change, fight stigma, and educate about recovery and policy change. It was clear that many of our participants wanted to use their collections of narratives to change the world.

Supporting others on their recovery journey was a goal of participants. The narrative has the potential to heal and connect people as well as shift paradigms,^20^ and receiving just a single narrative can cause a “reference shift” for a recipient, i.e., “a fundamental change in belief or understanding about the possibility of recovery, and how it might come about”.^21^ One participant revealed a wish to reframe mental illness, which is an idea supported by Mad studies.^22^ Mad studies considers the psycho–political sides of mental distress and challenges the concept of diagnostic labels.^23^

Lived experience narratives could be used to extend clinical practice.^24^ Another key goal of curators is to support others in their recovery, which is key in the work of peer support workers who use their own narratives in their work.^25^ Peer support worker training places a large emphasis on learning how to positively use personal recovery narratives to help to establish mutuality and reciprocity within peer relationships.^26^ This process of exploring the safe sharing of lived experiences can be enhanced and supported using recovery narrative collections.

### Limitations

Two limitations can be identified. Snowball sampling was started in the UK, and hence 60% of participants came from this country. Second, apart from one collection, only one curator per collection was interviewed. Interviews with multiple members of a curatorial team might reveal contrasting or even conflicting views on the process of curation for that collection.^11^

### Implications

Future curators would find this work useful in order for them to identify their goals as well as understand the possible implications of these on their curatorial practice. This work can help curators reflect about the outcome they would like to achieve with curating a collection and help individuals who are interested in curating a mental health recovery collection to reflect on why they may want to curate a collection. Knowledge and awareness of the goals of others might be seen as an essential antecedent to effective reflective practice^27^ in the curation of narrative collections, and reflective practice is already a core concern in museology,^28^ where it has found a place in professional training.^29^ As such, our results might shape future training for curators of recovery narrative collections and aid in its development as a professional practice.

Sharing a narrative can provide benefits for narrators,^30^ and some narrators have used storytelling as a political tool to challenge psychiatry.^31^ Understanding that the goals of curators can have a powerful influence on the contents of narrative collections might be important for people wanting to share their narratives and might shape a decision about whether to choose a particular collection or not.^32^ To enable a narrator to make a decision that works for them, collections might need to be explicit about the goals that underpin them, and future narrators might be advised to assess whether the goals of a collection fit with their personal goals. Transparency of inclusion criteria and processes for editing, withdrawal, and anonymizing narratives might also be considered important in enabling narrator decision-making.^33^ A collection of narratives can be a powerful evidence base that could be used for policy change,^6^ and transparency of goals and process might allow for more effective policy-making, in that it can make manifest the various factors that influenced the collection. Curators wishing to influence policy-making might choose to prioritize transparency so as to maximize their impact on policy-making.

A collection of narratives can be created for use by activists for campaigning^34^ or antistigma work.^35^ Activists might consider the existing ways of working with narratives described in this paper and identify ways of working with narratives that might better promote their campaigns. Further research might collect evidence for how narrative collections as a form of activism have made an impact so as to further develop the evidence base for the use of collections in activism.

## Figures and Tables

**Table 1:** Sociodemographic Characteristics of Participants (N=30)

Characteristic		n (%)
**Gender**	Female	19 (63%)
	Male	11 (37%)
	Prefer to self-describe	0 (%)
	Prefer not to say	0 (%)
**Ethnicity**	White	24 (80%)
	Asian	3 (10%)
	Black	1 (3%)
	Mixed or multiple ethnic groups	1 (3%)
	Prefer to self-describe	1 (3%)
**Age**	Under 25	2 (7%)
	25 - 34	4 (13%)
	35 - 44	13 (43%)
	45 - 54	5 (17%)
	55 - 64	5 (17%)
	Above 64	1 (3%)
**Country of origin**	UK	18 (60%)
	USA	6 (20%)
	Canada	2 (7%)
	Brazil	1 (3%)
	Hong Kong	1 (3%)
	India	1 (3%)
	Italy	1 (3%)
**Lived experience of mental health service use**	No experience	12 (40%)
	Primary care	1 (3%)
	Secondary care	6 (20%)
	Voluntary hospitalization	8 (27%)
	Involuntary hospitalization	3 (10%)
**Sexual orientation**	Heterosexual	22 (73%)
	LGBTQ +	4 (13%)
	Prefer to self-describe	2 (7%)
	Prefer not to say	2 (7%)
**Educational level**	Higher degree	23 (77%)
	Bachelor’s degree	7 (23%)
**Employment status at time of curating**	Full-time paid employment	18 (60%)
	Part-time paid employment	5 (17%)
	Self-employed	2 (6%)
	Student	2 (6%)
	Volunteer	2 (6%)
	Retired	1 (3%)
	No	23 (77%)
**Disability as declared by the participant**	Yes	7 (23%)
**Income**	Below GB £10,000	4 (13%)
	GB £10,000 - GB £15,000	1 (3%)
	GB £15,001 - GB £20,000	3 (10%)
	GB £20,001 - GB £35,000	6 (20%)
	GB £35,001 - GB £50,000	6 (20%)
	GB £50,001 - GB £100,000	5 (17%)
	Above GB £100,000	1 (3%)
	Prefer not to say	2 (6%)

**Table 2:** Goals of Curators

Goal	n (% of total participants)
Fighting stigma	11 (37%)
Campaigning for change in service provision	9 (30%)
Educating about mental health and recovery	9 (30%)
Supporting others in their recovery journey	8 (27%)
Critiquing psychiatry	5 (17%)
Influencing policy change	2 (7%)
Marketing health services	1 (3%)
Reframing mental illness	1 (3%)

**Table 3:** Curatorial Practices Influenced by Goals of Participant

Curatorial practice	Specific decision influenced
Selection and inclusion	Selecting narratives with a specific sociopolitical standpoint e.g., to fit within the message of a campaign Selecting narratives with specific trajectories (e.g., the narrative ends hopefully) Selecting narratives describing specific types of mental health experience
Editing	Editing content to match the goal (e.g., to promote an antistigma message)
Withdrawal	Not allowing withdrawal of narratives
Anonymization	Not allowing anonymization of narrators
